# Interferon-λ in HCV Infection and Therapy

**DOI:** 10.3390/v2081589

**Published:** 2010-08-05

**Authors:** Nicole E. Pagliaccetti, Michael D. Robek

**Affiliations:** Department of Pathology, Yale University School of Medicine, New Haven, CT 06510, USA; E-Mail: nicole.pagliaccetti@yale.edu

**Keywords:** IFN-λ, IL-28, IL-29, type III interferon, *IL28B*, IL-28Rα

## Abstract

Chronic infection with hepatitis C virus (HCV) is associated with significant liver disease and is therefore an important public health problem. The current standard-of-care therapy for chronic HCV infection consists of a combination of pegylated (PEG) interferon (IFN)-α and ribavirin. Although this therapy effectively generates a sustained viral response in approximately half of treated individuals, it is associated with significant hematological and neurological side effects. A new family of IFN-related proteins (IFN-λ1, 2, and 3; or alternately, IL-29, 28A, 28B, respectively) possesses properties that may make these cytokines superior to PEG-IFN-α for HCV therapy. Genetic studies have also implicated these proteins in both the natural and therapy-induced resolution of HCV infection. This review summarizes the basic aspects of IFN-λ biology, the potential role of these cytokines in HCV infection, and the outlook for their therapeutic application.

## 1. Structural properties of IFN-λ

The IFN-λ family of IFN-related proteins was discovered in 2003 using computational methods designed to find new proteins within the class II α-helical cytokine family [1,2]. Three members of this family were identified and alternatively named IFN-λ1, 2, 3, or IL-29, 28A, 28B, and are now also referred to as the “type III” IFNs to further distinguish them from IFN-α/β (type I) and IFN-γ (type II). Although the three IFN-λ family members have a high degree of amino acid identity to each other (81% between IFN-λ1 and IFN-λ2; 96% between IFN-λ2 and IFN-λ3), these proteins have low sequence homology to both IFN-α (15–19% identity, 31–33% similarity) and IL-10 (11–13% identity, 22–23% similarity) [1–3]. Despite this minimal homology, conserved cysteine patterns and predicted amphipathic helix profiles indicated that the IFN-λs belong to the class II cytokine family. Furthermore, the IFN-λ genes are composed of five to six exons, an arrangement that is similar to IL-10, but is unlike the type I interferons, which are each encoded by a single exon [1,2]. Recently, the crystal structure of IFN-λ3 was reported to contain a bundle of four alpha helices at its core, which is similar to other class II cytokines [3]. Further comparison of the structure between IFN-λ and other class II cytokines found a closer association between IFN-λ and IL-10 family cytokines, in particular IL-22, than with the type I IFNs. Therefore, despite low amino acid homology between IFN-λ and the IL-10 family cytokines, there is a strong structural correlation between these two groups of proteins.

The IFN-λs do not bind to the IFN-α/β receptor, but instead exert their activity through a distinct receptor. The IFN-λ receptor consists of two subunits: IL-28Rα and IL-10Rβ [1,2]. The IL-10Rβ subunit is not unique to IFN-λ, as it is also utilized by IL-10 and IL-22 [4,5]. While the IL-10Rβ subunit is ubiquitously expressed on many cell types [6], the IL-28Rα subunit displays a more restricted profile [1,2], and is most strongly expressed on cells of epithelial origin [7–10]. Although the regulation of IFN-λ expression has been well studied (described in section 2 below), there is little known about the mechanisms that control expression of the IL-28Rα receptor subunit. Since the expression of the IFN-λ receptor is highly dependent on cell type, there may be tissue-specific signals required to induce IL-28Rα expression, such as specific transcription factors, DNA and histone methylation patterns, or microRNAs. Interestingly, it was also recently found that a splice variant of the IL-28Rα transcript encodes a soluble version of the receptor that inhibits IFN-λ activity in leukocytes [8], further indicating that precise restriction of IFN-λ activity to specific cell types may be important for its biological function.

## 2. Activation of IFN-λ expression

IFN-λ expression has been detected in primary neuronal cells, alveolar epithelial cells, hepatocytes, and a variety of cell lines [7,11,12]. However, like IFN-α, the primary producers of IFN-λ appear to be dendritic cells (DCs) [7,11–14]. Similar to the type I IFNs, IFN-λ expression is induced following viral infection or activation of Toll-like receptors (TLRs). Stimulation of the cytoplasmic receptor RIG-I, which detects cytoplasmic viral RNA, activates IFN-λ expression [15,16]. While many cell types may produce IFN-λ following TLR activation, DCs and DC-derived cell lines are the best characterized, as they produce relatively high levels of IFN-λ [12,14,15,17]. Activation of TLRs-3, -4, -7 and -9 all increase IFN-λ expression in DCs [14,15]. TLR-3, -7 and -9 are typically localized in endosomes and detect viral pathogen-associated molecular patterns such as doubled stranded RNA (dsRNA), single stranded RNA, and non-methylated double-stranded CpG-rich DNA [18]. Stimulation of these receptors ultimately leads to the activation of transcription factors such as interferon-regulatory factor (IRF)-3, IRF-7 and NF-κB. Though IFN-λ is typically activated by viral infections, activation of TLR-4 by bacterial LPS has been shown to induce IFN-λ in DCs [14,19], suggesting that IFN-λ may have additional functions in the modulation of the immune response [19].

Binding sites for IRF-3, IRF-7, and NF-κB have all been identified in the promoter region for IFN-λ and are essential for induction of expression [16]. However, there are differences in the regulation of IFN-λ1 and IFN-λ2/3 transcription. IFN-λ1 is activated by both IRF-3 and -7, whereas IFN-λ2/3 is primarily regulated by IRF-7 [20]. In contrast to the type I IFNs, which are not induced by IFN treatment, IFN-λ mRNA expression can be induced by stimulating cells with IFN-α or IFN-λ alone, indicating that IFN-λ is in fact also an interferon-stimulated gene (ISG) [21]. Furthermore, IFN-α may play a role in regulating TLR-induced activation of IFN-λ expression [22], demonstrating additional cross-talk between the type I and type III interferon responses.

The extent to which IFN-λ is induced in a natural HCV infection is unclear, as HCV has evolved multiple mechanisms to inhibit the IFN-α/β response in infected hepatocytes. The HCV NS3/4A protease inhibits IRF-3 activation and cleaves the RIG-I and TLR signaling adapters IPS-1 and TRIF [23–25]. A second HCV protein (NS2) also blocks activation of IFN-β through an uncharacterized mechanism that is distinct from that of NS3/4A [26], and the HCV NS5A protein is yet another factor capable of inhibiting IFN-α/β expression [27]. Because IFN-α/β and IFN-λ are activated through a common molecular mechanism [16] by identical types of stimuli [14], the viral immunomodulatory mechanisms that HCV has evolved to inhibit IFN-α/β expression also likely block IFN-λ production. In fact, when over-expressed in cell culture, NS3/4A prevents the induction of both IFN-α/β as well as IFN-λ [26]. Furthermore, like IFN-α/β, IFN-λ is expressed in PBMC but not in the liver of chronic HCV patients [28].

## 3. IFN-λ-induced signaling and gene expression

**Figure 2.**
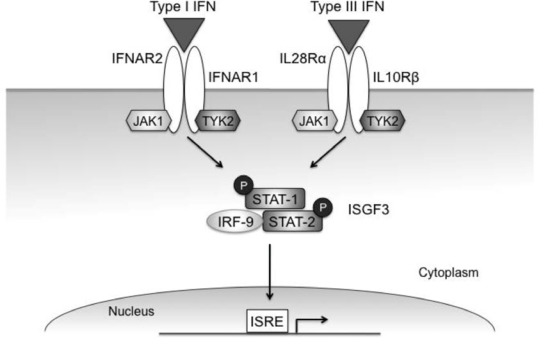
**Type I and type III IFN signaling pathways.** Though the type I and type III receptors are distinct, both cytokines induce STAT phosphorylation through the Jak kinases associated with the respective receptor subunits. Both IFN-α and IFN-λ primarily activate STAT-1 and -2, which complex with IRF-9 to form the transcription factor ISGF-3. This complex induces expression of genes with ISREs in their promoters.

The signaling pathways induced by IFN-λ are very similar to those induced by the type I IFNs (Figure 2) [29]. The intracellular domains of the IFN-λ receptor subunits, IL-28Rα and IL-10Rβ, interact with the receptor-associated tyrosine kinases Jak1 and Tyk2. These kinases in turn phosphorylate STAT proteins, which then dimerize and act as transcription factors. Binding of IFN-λ to its receptor induces phosphorylation of STAT-1, -2, -3, and -5 through a process that requires two key tyrosine residues on IL-28Rα [1,29–34]. In most cell types, IFN-λ induces phosphorylation of STAT-1 and STAT-2, which form a heterodimer that interacts with IRF-9 to form the transcription factor interferon-stimulated gene factor-3 (ISGF-3) [35]. ISGF-3 preferentially binds to promoters containing ISREs, which are found in the upstream regions of ISGs. Like IFN-α, IFN-λ-induced STAT activation is negatively regulated by the suppressor of cytokine signaling (SOCS) proteins [36].

Though the signaling pathway induced by IFN-λ is nearly identical to that of IFN-α, the kinetics and magnitude of the responses can be subtly different. In Huh-7 hepatocellular carcinoma cells, IFN-λ induces STAT-1 and STAT-2 more rapidly than IFN-α [30]. Additionally, IFN-λ induces STAT-1 and STAT-2 phosphorylation for a longer period of time in HaCaT keratinocytes compared to IFN-α [37]. Furthermore, although the subsequent transcriptional response is slightly delayed, the increase in ISG expression induced by IFN-λ is stronger and more prolonged than the response activated by IFN-α [30,37]. Nevertheless, with their signaling patterns being nearly identical, IFN-α and IFN-λ induce very similar patterns of gene expression [30,31,38]. Consistent with the convergence of the two signaling pathways to a similar transcriptional response, combinations of IFN-λ and IFN-α together appear to have no more than a dose-dependent additive effect on HCV replication [30,39].

Although Jak/STAT signaling mediates the primary functions of IFN-λ, other pathways are also activated by the receptor. One study found that IFN-λ activated ERK-1/2, mitogen activated protein kinase (MAPK) and Akt in intestinal epithelial and colorectal cancer-derived cell lines [40]. This activity led to increased IL-8 expression, a chemokine that is associated with the inflammatory response [41]. Additionally, activation of MAPKs was also observed in Raji cells following treatment with IFN-λ [38]. These results indicate that IFN-λ can induce multiple signaling pathways that may contribute to its activity as an antiviral and immunomodulatory cytokine.

## 4. Functions of IFN-λ

### 4.1. IFN-λ antiviral activity

Much of the early work on IFN-λ was devoted to determining its ability to inhibit virus replication in cell culture model systems. Encephalomyocarditis virus (EMCV), vesicular stomatitis virus, cytomegalovirus, herpes simplex virus 1, influenza A virus, HIV, HBV, and HCV are all sensitive to the antiviral effects of IFN-λ [1,2,21,30,31,42–47]. Specifically with respect to HCV replication, antiviral activity of IFN-λ has been demonstrated using both replicon (subgenomic and full-length genomic) and cell culture infectious virus model systems [30,31,39,43,45] in Huh-7 hepatocellular carcinoma cells. Similarly to HCV, HBV also is inhibited by IFN-λ in mouse immortalized hepatocytes, and to a somewhat lesser extent, in the human hepatoblastoma cell line HepG2 [31,45,48]. Therefore, IFN-λ has clear antiviral activity against human hepatotropic viruses.

For some viruses, infection, but not preexisting replication, is inhibited by IFN-λ. IFN-λ prevents West Nile virus infection, but not the replication of virus-like particles in a hepatocyte derived cell line [49]. Similarly, IFN-λ prevents Hantavirus infection of lung epithelial cells *in vitro*, but it is unable to inhibit replication once an infection is established [50]. Furthermore, IFN-λ does not inhibit the replication of Lassa virus in macrophages or DCs [51]. It should be noted however that neither IFN-α nor IFN-γ inhibited Hantavirus infection, and IFN-γ was also unable to prevent Lassa virus replication in the reported studies. Therefore, the inability of IFN-λ to inhibit these viruses may be due to their inherent insensitivity to the antiviral effects of IFNs in general, rather than a specific shortcoming of the IFN-λ response.

Compared to cell culture studies on the antiviral activity of IFN-λ, the number of reports on IFN-λ activity in animal models is relatively limited. *In vivo* antiviral activity of IFN-λ in mice appears to be highly dependent on the virus and the administration route. Intraperitoneal administration of IFN-λ2 protects mice from HSV-2 infection in the liver, but intravenous injection of IFN-λ2 does not provide protection from EMCV or lymphocytic choriomeningitis virus in the heart or spleen, respectively [21]. Similarly, intraperitoneal injection of IFN-λ3 does not protect mice from infection by the hepatotropic virus Thogotovirus, but intranasal administration of IFN-λ3 confers protection from influenza A virus infection in the lungs [9]. In mice, cellular sensitivity to IFN-λ correlates strongly with expression of the IL-28Rα receptor subunit, which is most prominently expressed in epithelial cells of the gastrointestinal and respiratory tracts [7,10].

An alternative delivery method for IFN-λ was utilized by Bartlett and colleagues to test the activity of IFN-λ against poxviruses in mice. Rather than administer the cytokine systemically, a recombinant vaccinia virus (VACV) was engineered to express murine IFN-λ2 or IFN-λ3, and mice were infected with the virus intranasally or intradermally [52]. VACV expressing IFN-λ did not cause any symptoms in infected mice and was cleared more rapidly compared to the control viruses after intranasal infection. IFN-λ also limited the infection of VACV after intradermal infection, as the lesions caused by the infection were both delayed in appearance and reduced in size [52]. However, IFN-λ does not directly inhibit VACV replication in cell culture, indicating that these effects are likely due to immunomodulatory, rather than antiviral, activities of the cytokine [52,53].

### 4.2. IFN-λ immunomodulatory activity

IFN-λ clearly has direct inhibitory effects on the replication of most viruses, and therefore may be an important component of the innate immune response, at least in certain contexts. However, a number of studies have also demonstrated that IFN-λ also plays a role in antiviral immunity through modulation of both the maturation and differentiation of immune cells. Though monocytes express low levels of the IFN-λ receptor, differentiation of these cells into DCs leads to an upregulation of IL-28Rα and an increased ability to express IFN-λ [54–56]. Conversely, monocytes that differentiate into macrophages show reduced expression of IFN-λ [56]. Additionally, when DCs are subsequently exposed to IFN-λ, increased maturation and migration capacity are induced [55]. These changes are due in large part to changes of cell surface molecules on DCs that alter both the stimulation and homing of these antigen-presenting cells [54]. In turn, IFN-λ influences the effects that DCs have when interacting with T cells. DCs treated with IFN-λ preferentially expand regulatory T cells, which are critical for negative regulation of the immune response, as well as promoting self-tolerance [55].

By altering the maturation and differentiation of other immune cells, particularly T cells, IFN-λ also alters the expression of other cytokines and chemokines. The role of IFN-λ appears to be primarily focused on biasing T cell differentiation against Th2 development and Th2 cytokine secretion [57,58]. IFN-λ inhibits IL-4, IL-5 and IL-13 expression in T cells independently of IL-10 [58,59], and modulates both cytokine and chemokine expression in peripheral blood mononuclear cells (PBMCs), reducing IL-13 as well as IL-6, IL-8 and IL-10 production [60]. Additionally, the chemokines MIG, IP-10 and I-TAC, which are antimicrobial chemoattractants for mononuclear cells, were found to be upregulated by IFN-λ in PBMCs [61]. In total, these studies indicate a potential important role for IFN-λ in both the regulation and development of the adaptive immune response.

## 5. *IL28B* polymorphisms and HCV infection/therapy outcome

Recent genome-wide association studies have found a strong genetic link between HCV infection, treatment outcome, and IFN-λ. Both spontaneous HCV clearance and a sustained viral response following PEG-IFN-α plus ribavirin therapy correlate with single nucleotide polymorphisms (SNPs) found in the *IL28B* gene locus, which encodes the IFN-λ3 protein [62–66]. The rs12979860 SNP resides 3 kb upstream of the *IL28B* gene, and variations at this position are associated with approximately 2-fold differences in spontaneous clearance and response to treatment [62–64]. The C/C genotype is associated with better outcomes, and the T/T genotype, worse outcomes. The rs8099917 SNP is located within an intergenic region between the *IL28A* and *IL28B* genes, and is similarly associated with a 2–3 fold difference in spontaneous clearance and response to therapy [65–67]. The polymorphisms associated with poor response to therapy are found at a higher frequency in African populations compared to European populations, consistent with the lower response rates of PEG-IFN-α plus ribavirin treatment in African-Americans [68].

Although the relationship between HCV infection outcome and therapy response with *IL28B* variation is now well established, the molecular mechanisms behind this association have not yet been identified. The rs12979860 SNP is associated with two other polymorphisms found in the IFN-λ3 transcription initiation and coding regions, which may potentially alter the expression or activity of the cytokine [65,66]. Individuals harboring the rs8099917 minor allele were found to have reduced IFN-λ3 expression levels in PBMC, indicating that this variant may be located within a transcriptional regulatory element [63,64]. Because IFN-α upregulates IFN-λ expression [21,22], IFN-λ may amplify interferon-stimulated gene expression following administration of PEG-IFN-α. As other host factors have also been associated with therapy outcome, the interplay between these various factors needs to be better defined. For example, patients who fail to achieve a sustained viral response after PEG-IFN-α therapy have a high pre-therapy level of intrahepatic ISG expression [69,70], and it has not yet been addressed whether this observation is also related to *IL28B* variation. Elucidation of these mechanisms will be important for understanding the role of IFN-λ in chronic HCV infection and in IFN-based therapies.

## 6. IFN-λ and HCV Therapy

It was immediately recognized after its discovery that due to the relatively restricted expression of the IFN-λ receptor, the type III IFNs could potentially be useful therapeutically for chronic HCV infection [1,2]. Because the receptor is expressed at low levels on T cells and NK cells, and is not expressed on hematopoetic precursor cells, it was predicted that therapeutic use of IFN-λ would not cause the hematological side effects associated with PEG-IFN-α therapy [71]. This prediction has largely been proven true by a recently reported Phase 1b clinical trial of PEG-IFN-λ1 for genotype 1 chronic HCV infection [72]. In this study, the antiviral efficacy and side effects of PEG-IFN-λ1 were measured in IFN-α relapse patients as a single agent, and in relapse and treatment-naïve patients in combination with ribavirin.

The majority of patients in this study achieved significant reductions in HCV RNA levels after 4 weeks of treatment with PEG-IFN-λ1, both as a single agent alone and when administered together with ribavirin. More specifically, in individuals who received weekly doses of 1.5 μg/kg or greater, 96% (23 of 24) of treatment-relapse patients and 86% (6 of 7) of treatment-naïve patients had a > 2 log_10_ decline in viral RNA [72]. Furthermore, a subset of these patients (17% of relapse and 29% of naïve) attained undetectable levels of HCV RNA. While not directly compared in this study, the biphasic kinetics of viral decline were found to be similar to the pattern typically observed with IFN-α therapy. This study also demonstrated that PEG-IFN-λ1 administration did not cause the significant reductions in neutrophil counts, platelet counts, or hemoglobin levels that can be associated with PEG-IFN-α therapy [73]. IFN-λ1 therapy was generally otherwise well-tolerated, with adverse events such as fatigue, nausea, myalgia, fever, or irritability being relatively rare and mild [71,72]. Therefore, PEG-IFN-λ1 may have fewer of the safety and tolerability issues that can limit PEG-IFN-α efficacy.

While the results from the Phase 1 trial are very promising, a number of questions remain regarding the clinical utility of IFN-λ for chronic HCV that can only be resolved through additional larger studies. First, will IFN-λ therapy successfully generate a long-term sustained viral response after cessation of therapy? Second, how will the response to IFN-λ be influenced by the *IL28B* polymorphisms that affect PEG-IFN-α therapy? Third, will the efficacy of IFN-λ1 extend to HCV genotypes other than genotype 1? Fourth, will long-term IFN-λ administration cause the same neurological side effects that can accompany IFN-α therapy? Despite these unresolved questions, the encouraging results obtained thus far give reason to be optimistic that the clinical potential of IFN-λ will be realized not only for chronic HCV infection, but also for other diseases that respond to IFN-α therapy, such as chronic HBV infection and melanoma.

## Figures and Tables

**Figure 1.**
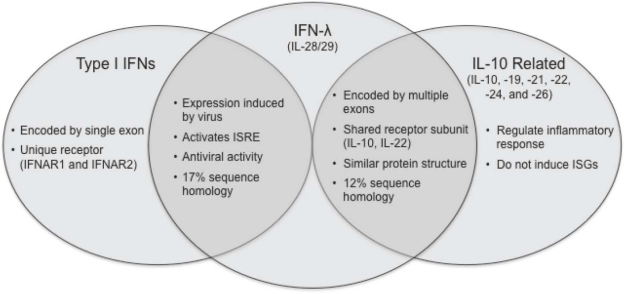
**Comparison of type I IFNs, IFN-λ and IL-10 related cytokines.** The type III IFNs are functionally similar to type I IFNs (IFN-α/β). However, they are more structurally related to the IL-10 family cytokines.
